# The Anti-Cancer Property of Proteins Extracted from *Gynura procumbens* (Lour.) Merr

**DOI:** 10.1371/journal.pone.0068524

**Published:** 2013-07-11

**Authors:** Chaw-Sen Hew, Boon-Yin Khoo, Lay-Harn Gam

**Affiliations:** 1 School of Pharmaceutical Sciences, Universiti Sains Malaysia, Penang, Malaysia; 2 Institute for Research in Molecular Medicine (INFORMM), Universiti Sains Malaysia, Penang, Malaysia; University of Oklahoma Health Sciences Center, United States of America

## Abstract

*Gynura procumbens* (Lour.) Merr. belongs to the Asteraceae Family. The plant is a well-known traditional herb in South East Asia and it is widely used to treat inflammation, kidney discomfort, high cholesterol level, diabetic, cancer and high blood pressure. Our earlier study showed the presence of valuable plant defense proteins, such as peroxidase, thaumatin-like proteins and miraculin in the leaf of *G. procumbens*. However, the effects of these defense proteins on cancers have never been determined previously. In the present study, we investigated the bioactivity of gel filtration fractionated proteins of *G. procumbens* leaf extract. The active protein fraction, SN-F11/12, was found to inhibit the growth of a breast cancer cell line, MDA-MB-231, at an EC50 value of 3.8 µg/mL. The mRNA expressions of proliferation markers, Ki67 and PCNA, were reduced significantly in the MDA-MB-23 cells treated with SN-F11/12. The expression of invasion marker, CCL2, was also found reduced in the treated MDA-MB-231 cells. All these findings highlight the anti-cancer property of SN-F11/12, therefore, the proteins in this fraction can be a potential chemotherapeutic agent for breast cancer treatment.

## Introduction

Many of the currently available drugs are plant-based, and plant peptides and proteins have turned out to be a critical source of biological compounds that exhibited bioactivities which can be exploited as drug. Amongst the activities being discovered were the anti-tumor activity of peptides extracted from *Hypericum perforatum, Chelidonium majus L*., *Inula helenium L*., *Equiseteum arvense L*., and *Inonotus obliquus* [1]; anti-HIV property of macrocyclic peptide extracted from *Palicourea* condensate [2]; anti-microbial activity of thaumatin-like proteins extracted from malting barley [3] and in different types of plants’ proteins [4]. The plant-based products including proteins and small molecular compounds have been suggested as the favorable drugs for cancer treatment in view of the many adverse effects exerted by current cancer treatments, namely chemotherapy and radiation therapy. Moreover, these treatments are costly, which may be a burden to the patients. Plants contain various active ingredients that can be used as medicine for treatments of diseases such as breast cancer [5]. Breast cancer is the leading cause of death due to cancer among females worldwide [6]. Although radiation therapy and chemotherapy are regular modes of treatment given to cancer patients, effective treatment for breast cancer remain limited. In light of this, significant effort to strive for effective agents from plant should be taken to identify more novel agents for both prevention and treatment of human cancers.

*Gynura procumbens* (Lour.) Merr. is a well-known traditional herb in South East Asia. The plant belongs to the Asteraceae Family. This plant is about 10–25 cm high and it is presented with succulent, elliptic and glossy purplish leaves [7]. The leaves of *G. procumbens* have been served as food for decades in Malaysia, where it is generally consumed raw as salad. Besides, the plant is widely used to treat inflammation, kidney discomfort, high cholesterol level, diabetic, cancer and high blood pressure [7], [8]. Indeed, *G. procumbens* is used traditionally in South East Asia for its valuable medicinal property. The small molecular weight compounds extracted from *G. procumbens* have been reported to display anti-cancer [9], anti-oxidant [10], anti-inflammatory [11], anti-hyperglycemic and anti-hyperlipidemic [12] activities. Moreover, we have detected the presence of valuable plant defense proteins, such as peroxidase, thaumatin-like proteins and miraculin from the leaf of *G. procumbens* [13],[14]. However, the effects of protein extract on cancers have never been determined. Therefore, we aimed to analyze the cytotoxic activity of the proteins extracted from the leaves of *G. procumbens* using lactate dehydrogenase (LDH) cytotoxicity assay and thereafter to determine the route of cytotoxicity mechanism through the expression of proliferation and invasion markers in the treated MDA-MB-231 cells using semi-quantitative polymerase chain reaction (PCR). Furthermore, active proteins were identified using mass spectrometric analysis. We hope that the data obtained will be useful for the future intervention of protein-based drug for cancer therapy.

## Materials and Methods

### Plant Materials

Leaves of *G. procumbens* were collected from Penang, Malaysia. No specific permissions were required for collection of the leaves and the plant is not a protected plant species. A voucher number of specimen (11209) was deposited at the Herbarium of the School of Biological Sciences, Universiti Sains Malaysia. Fresh leaves were washed twice with tap water and rinsed with distilled water.

### Protein Extract and Purification

Protein was extracted by the method of Kiba *et al.* (2003) [15] with slight modification. Fresh leaves of *G*. *procumbens* (1 kg) were ground to a fine powder form in liquid nitrogen using blender and mixed with 3 L of extraction buffer [10 mM NaH_2_PO_4_, 15 mM Na_2_HPO_4_, 100 mM potassium chloride, 2 mM ethylenediaminetetraacetic acid, 2 mM thiourea, 1 mM phenylmethanesulfonylfluoride and 1.5% (w/v) polyvinylpolypyrrolidone]. The mixture was homogenized every 20 min for 2 hr and incubated at 4°C overnight. The homogenate was centrifuged at 15 000 rpm, 4°C for 30 min. The crude extract was kept at −20°C. Two steps of ammonium sulfate precipitation were used, where solid ammonium sulfate was added to the crude extract of *G. procumbens* at 4°C until 30% saturation was achieved. The precipitate was removed by centrifugation for 30 min at 13 000 rpm. Subsequently, solid ammonium sulfate was added again to the remaining supernatant to achieve 70% saturation and the solution was centrifuged for 30 min at 13 000 rpm and the pellet was recovered. The pellet was then resuspended in deionized water until fully dissolved. The crude ammonium sulfate precipitated proteins was loaded to a gel filtration column, HiPrep™ 16/60 Sephacryl™ S-100 High Resolution (GE healthcare) using ÄKTAprime plus protein purification system. Proteins were eluted with 50 mM sodium phosphate, pH7.2 at a flow rate 0.5 mL/min for a total volume of 180 mL elution buffer, the eluate was collected in the fractions of 5 mL each. The concentration of protein was determined using RC/DC™ Protein Assay Kit (Bio-Rad Laboratories).

### SDS-PAGE

Sodium dodecyl sulphate polyacrylamide gel electrophoresis (SDS-PAGE) was used to separate the collected proteins. Each fraction was mixed with non-reduce sample buffer [0.5 M Tris-HCl (pH 6.8), 10% (v/v) glycerol, 0.02% (w/v) SDS and 0.1% (w/v) Bromophenol Blue]. Electrophoresis process was run at a constant voltage of 200V. The gel was stained with Coomassie Blue.

### LDH Cytotoxicity Assay

The MDA-MB-231 (HTB-26™) cell line was a kind gift from John Hopkins Research Centre, which was purchased from American Type Culture Collection (ATCC). The cells were cultured in Dulbecco’s Modified Eagle Medium (DMEM) (Gibco BRL) supplemented with 10% (v/v) fetal bovine serum (FBS) (Gibco BRL), 100 Units/mL penicillin and 100 mg/mL streptomycin (Gibco BRL). The cells were incubated at 37°C in a humidified atmosphere that consisted of 5% (v/v) CO_2_. The cytotoxic activity was performed using lactate dehydrogenase (LDH) cytotoxicity assay kit (BioVision, USA), LDH being a cytoplasmic enzyme present in all cells, so it will be released into the culture medium when damage of the cell plasma membrane take place. LDH activity was determined by a coupled enzymatic reaction where LDH oxidized lactate to pyruvate, the pyruvate formed then reacted with tetrazolium salt INT to form farmazan. The increase in the amount of formazan is directly correlated to the increase in the number of lysed cells. The formazan dye intensity was measured by an ELISA reader. A total of 1.0×10^5^ cells/mL MDA-MB-231 cells was seeded into 24-well culture plate which was incubated at 37°C in a humidified atmosphere consisting of 5% (v/v) CO_2_ until the cell growth achieved approximately 70% confluency. The medium from each well was discarded, the cells were gently washed with PBS and 2% of growth medium (DMEM supplemented with 2% (v/v) FBS, 100 Units/mL penicillin and 100 mg/mL streptomycin) was added. The cells were treated with increasing concentrations of the extract (triplicate). One hundred µL of culture medium was withdrawn and LDH cytotoxicity assay was performed according to the manual provided. One hundred µL of culture medium were transferred into an immuno plate and 100 µL reaction mixture (mixture of catalyst and dye solution) was added to each well. The plate was incubated for 30 min at room temperature in the dark. The absorbance of all the samples was measured at 490 nm and the reference wavelength was 680 nm. The percentage of cytotoxicity was calculated as [(test sample – low control)/(high control – low control)]x100, where low control is the absorbance of culture medium without addition of any extract and high control is the absorbance of culture medium where the cells were treated with 1% (v/v) Triton X-100 to release 100% LDH.

### Polymerase Chain Reaction (PCR) Analysis

Total RNA was extracted from cells using the RNeasy Mini Kits (Qiagen, USA). The total RNA extracted was reverse transcribed to cDNA using the RevertAid First Strand cDNA Synthesis Kit (Thermo Scientific). The expressions of two proliferative markers, Ki-67 and PCNA, and two invasion markers, CCL2 and IL6, were examined in treated and un-treated MDA-MB-231 cells using PCR and gel densitometry. The levels of mRNAs were expressed as the band intensity of the markers of the PCR products relative to the control beta actin PCR product. The primers used for the PCR reactions were as follows: antigen Ki-67, Forward: 5′-AACTATCCTCGTCTGTCCCAACAC-3′, Reverse: 5′-CGGCCATTGGAAAGACAGAT-3′; proliferating cell nuclear antigen (PCNA), Forward: 5′-AGAAGGTGTTGGAGGCACTCA-3′, Reverse: 5′-GGTTTACACCGCTGGAGCTAA-3′; chemokine (c-c motif) ligand 2 (CCL2), Forward: 5′- GCTGTGATCTTCAAGACCATTGTG-3′, Reverse: 5′-AGTGAGTGTTCAAGTCTTCGGAGTT-3′; interleukin 6 (IL6), Forward: 5′-CCAGTACCCCCAGGAGAAGATT-3′, Reverse: 5′-CCGTCGAGGATGTACCGAAT-3′; beta actin, Forward: 5′-CATTGCCGACAGGATGCA-3′, Reverse: 5′-CCGATCCACACGGAGTACTTG-3′. The PCR reactions consisted of 5 µL of cDNA (5 ng), 12.5 µL of Master Mix, 1 µL of each 20 pmol forward and reverse primers and DNase/RNase-free water to a final volume of 25 µL. The reactions were set for 30 cycles with the following conditions; pre-amplification: 94°C for 10 min, denaturation: 94°C for 20 s, annealing: 55°C for 20 s, extension: 72°C for 30 s, termination: 72°C for 10 min. Each PCR reaction was carried out in triplicate and the PCR products were subjected to electrophoresis on 2% (w/v) agarose gel containing 0.1 µg/mL EtBr. Bands of PCR product on the gel were captured using the Gene Genius Image Analyser and the band intensity was analyzed using Quantity One software (Bio-Rad, USA).

### In-gel Digestion

In-gel digestion was performed using the method described by [16]. Protein bands were excised from SDS-PAGE. The gel pieces were washed with 100 mM ammonium bicarbonate for 10 min and then with acetonitrile (ACN) for 5 min. This step was repeated twice. Gel pieces were then dried in a speed-vacuum centrifuge. The dried gel pieces were added with 10 mM DTT in 100 mM ammonium bicarbonate and incubated for 1 hour at 56°C. Excessive solution was removed. The gel pieces were then incubated with 55 mM iodoacetic acid in 100 mM ammonium bicarbonate in the dark for 45 min at room temperature. The gel pieces were washed with 100 mM ammonium bicarbonate for 10 min and then with acetonitrile (ACN) for 5 min twice. The gel pieces were treated with 15 ng/µL of trypsin in digestion buffer [50 mM ammonium bicarbonate, 5 mM CaCl_2_] and incubated at 37°C overnight. The supernatant of the tryptic digest was collected and the remaining peptides were extracted 3 times in 5% (v/v) formic acid in 30∶70 of ddH2O: ACN. Supernatants were pooled and blow dried using nitrogen gas.

### Reverse Phase Capillary Chromatography and Mass Spectrometry

Reverse phase capillary chromatography (Waters Acquity Ultra Performance Liquid Chromatography [UPLC]) was coupled with quadrupole time-of-flight tandem mass spectrometry (Waters, Milford, MA, USA) connected to a LockSpary Exact Mass calibrator. Ionization source used was ESI (electrospray soft ionization). The sample was injected into the system through an autosampler. The samples were injected into a Trizaic UPLC nanoTile (Waters, Milford, MA, USA) consisted of a trapping column and a capillary column. The protein digest was pre-concentrated and desalted on the trapping column cartridge packed with 1.8 µm High Strength Silica (HSS) particle at a flow rate of 8*μ*L/min of 99% A for 3 minutes. The peptides were then eluted on a reversed phase capillary column (1.8 µm HSS particle, 85 µm × 100 mm) at a gradient mode from 3% B to 40% B in 30 min at a flow rate of 0.45 µL/min. Solvent A consisted of water with 0.1% formic acid, and solvent B consisted of acetonitrile with 0.1% (v/v) formic acid. The eluate was subjected to the MS system. All mass spectra were acquired using the Q-TOF mass spectrometer. The Q-TOF parameters were set as follows: positive mode; capillary, 3.2 kV; sampling cone, 28.0; extraction cone, 2.0; source temperature, 70°C; desolvation temperature, 200°C; cone gas flow, 40.0 L/h; desolvation gas flow, 600.0 L/h; scan time, 0.2 s. Spectra were recorded from *m/z* 2 to 1200. External calibration was applied to all data using [Glu1]-Fibrinopeptide B standard (Waters). Survey scan acquisition was done on-line with capillary chromatographic separation; an initial TOF-MS scan was acquired over the mass range of 2–1200 m/z each second, with switching criteria for MS to MS/MS that included ion intensity (100 counts/s) and charge state (+2 ). MS/MS of precursor ion selected was acquired over the mass range of 50–1600 m/z. The collision energy was 6 eV.

### Protein Identification

Database search was performed on the MS/MS data generated using the entire taxonomy order *Viridiplantae* under SwissProt database. Peptide tolerance and fragment mass tolerance were set at 0.1 bDa and 2 Da, respectively, and only one missed cleavage is allowed. Carbamidomethylated cysteine was set as fixed modification, while oxidized methionine was set as variable modification.

### Statistical Analysis

Data are presented as mean ± standard error (SE) of three indepent experiments and statistical analysis was done using Student’s t test. A value of p<0.05 was considered statistically significant.

## Results

**Figure 1:**
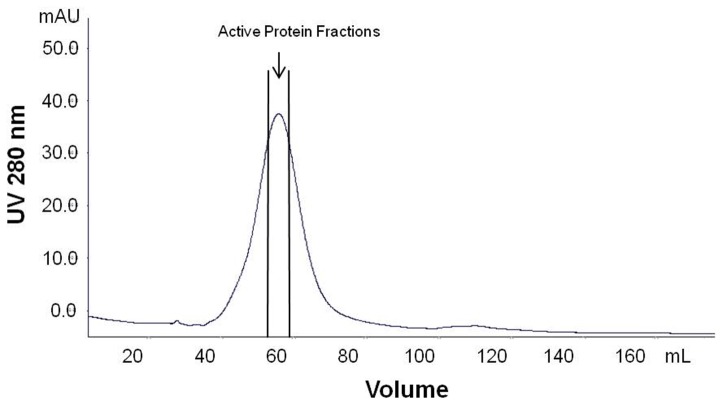
The chromatogram of crude protein extracted in the leaf of *G. procumbens*. The gel filtration separation was performed using HiPrep 16/60 Sephacryl S-100 HR column. The protein fractions were eluted with 50 mM sodium phosphate, pH 7.2, and flow rate of 0.5 mL/min. The active protein fractions 11 and 12 were eluted out within 50 mL to 60 mL of elution buffer.

**Figure 2:**
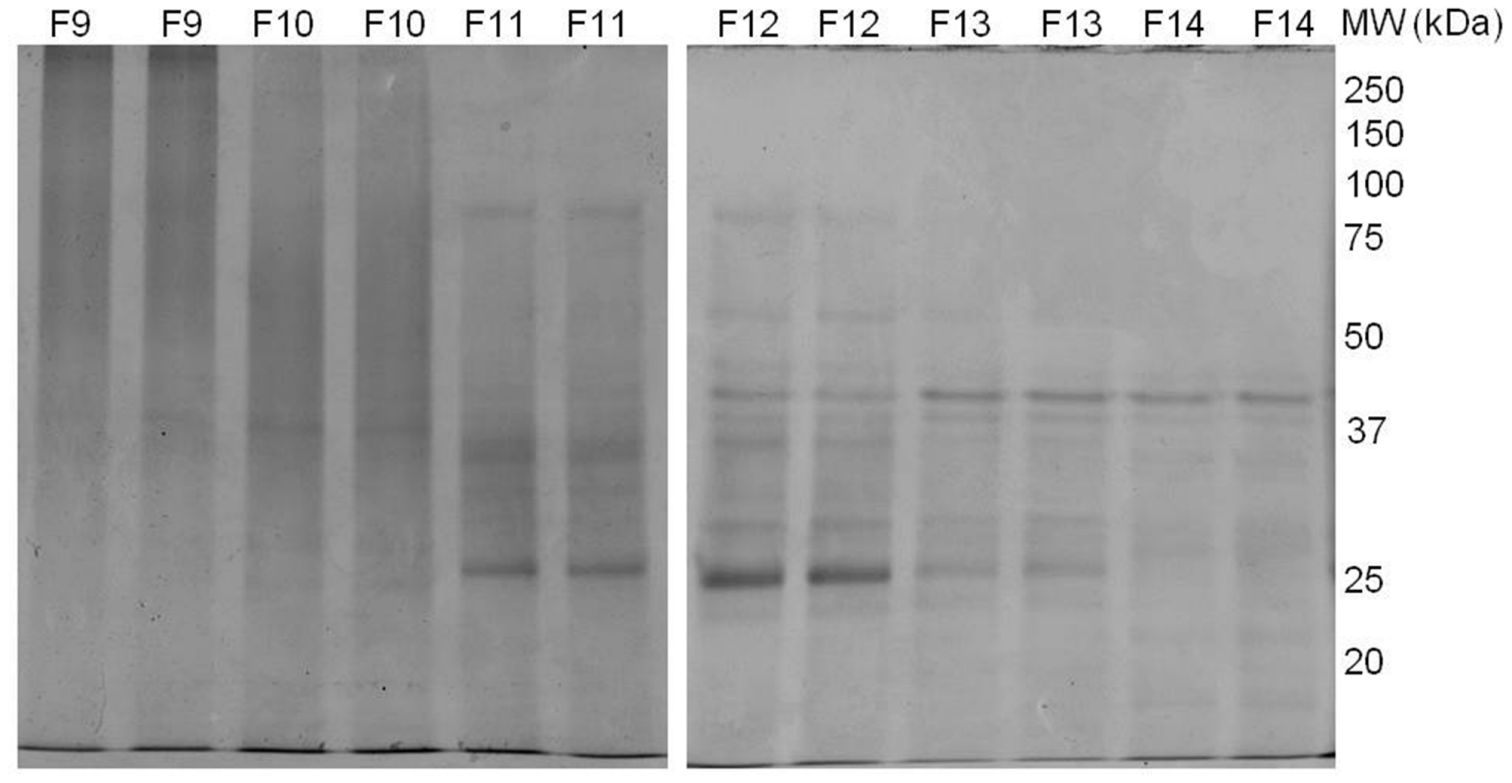
The SDS-PAGE analysis of protein fractions collected from gel filtration separation using HiPrep 16/60 Sephacryl S-100 HR column. Our preliminary analysis found that fractions 11 and 12 possessed anti-proliferation activity on MDA-MB-231. Both fractions 11 and 12 showed identical protein profiles.

Mild phosphate buffer was used to extract proteins from the leaf of *G. procumbens* in order to prevent denaturation of the proteins. *In vitro* bioactivity study indicated that the freeze-dried crude leaf extract of *G. procumbens* possessed anti-proliferation activity against the breast cancer cell line MDA-MB-231. Therefore, fractionation of the protein extract was performed in order to isolate the protein(s) of interest. Ammonium sulfate precipitation was employed as the first step in purification of the proteins as the method is non-denaturing to proteins, this salt precipitation method is aimed to remove small molecular weight compounds from the extract [17]. The salt precipitated proteins were then subjected to gel filtration, where further separation of proteins according to size was carried out. The eluate of gel filtration chromatography was collected in fractions of 5 mL volume each. All the fractions were tested for anti-proliferation activity against breast cancer cell line, MDA-MB-231 *in vitro*. Strong anti-proliferation activity was detected in fractions 11 and 12, where fraction 11 was consisted of the 51^st^ to 55^th^ mL eluate while fraction 12 was collected from the 56^th^ to 60^th^ mL eluate (Figure 1) from a total of 180 mL elution buffer used. Figure 2 shows the SDS-PAGE analysis for fractions 9 to 14.

**Figure 3:**
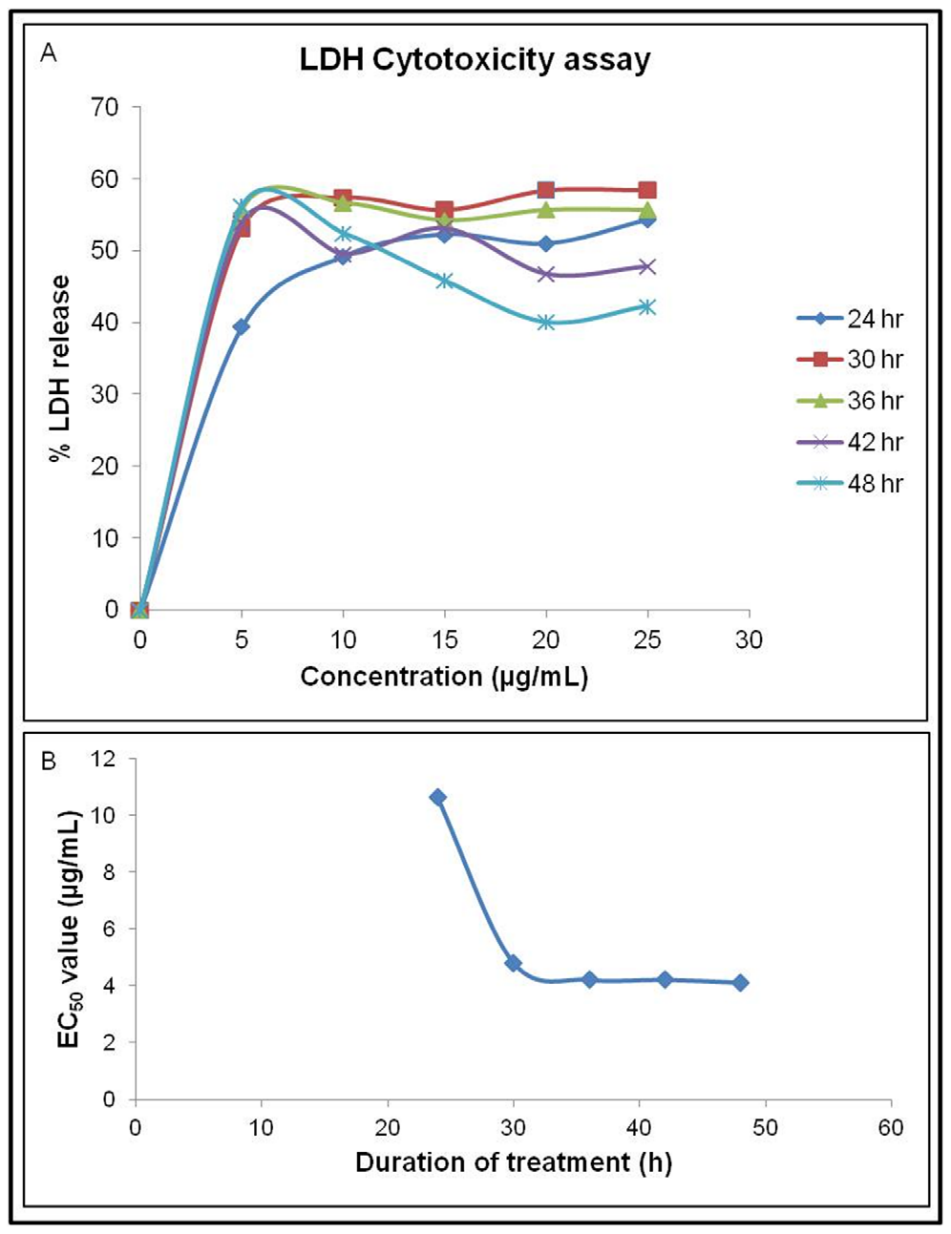
A. The dose- and time-dependent effects of SN-F11/12 on MDA-MB-231 using the LDH Cytotoxicity assay. Each curve of % LDH release was plotted against respective treatment time point. B. The EC50 value was plotted against respective treatment time point for the determination of constant EC50 value. The constant EC50 value of SN-F11/12 on MDA-MB-231 was 3.8 µg/mL.

Fractions 11 and 12 were pooled and termed SN-F11/12. LDH cytotoxicity assay was performed on the MDA-MB-231 cell line where the cell was treated with SN-F11/12. Figure 3a shows the percentage of LDH released into the culture mediums that were treated with different concentrations (5–25 µg/mL) of SN-F11/12, the treatments were carried out for 24, 30, 36, 42 and 48 hours and after each treatment period LDH percentage released to the mediums was measured. Effective concentration (EC_50_) value is defined as the concentration expressed in µg/mL of proteins in the extract that inhibited 50% of cell growth. EC_50_ values for different treatment periods were determined and are shown in Figure 3b, from which the constant EC_50_ value for SN-F11/12 was found to be at 3.8 µg/mL of proteins.

**Figure 4:**
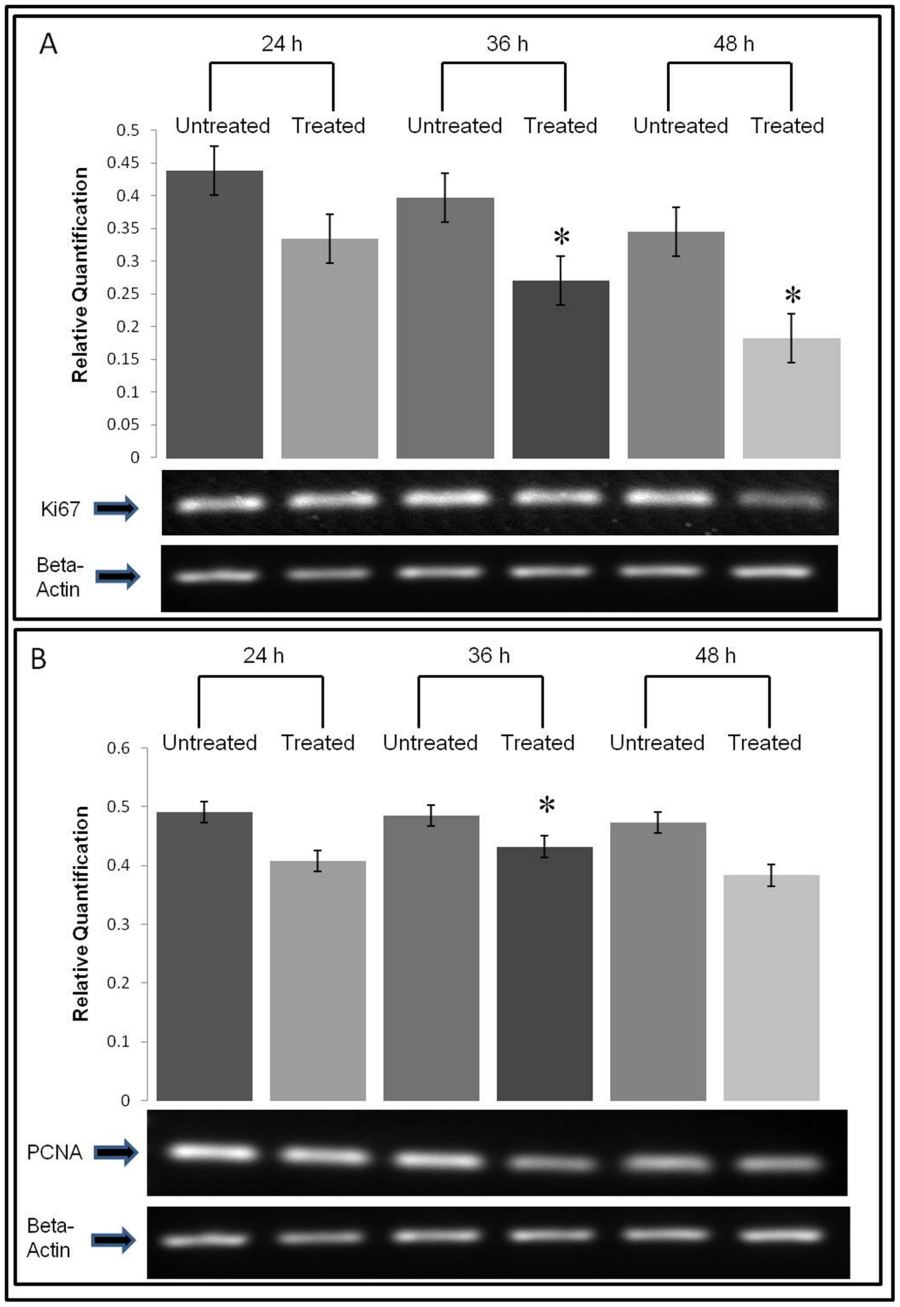
The expressions of proliferation markers on the SN-F11/12-treated MDA-MB-231 cells. The figures show normalised mRNA expression of (A) Ki67 and (B) PCNA on the treated MDA-MB-231 cells. The target gene mRNA expression was normalised to the mRNA expression of beta-actin. Each data represents mean ± SE from three independent experiments. Statistical analysis was determined using the Student’s *t* test with *p<0.05 as statistical significance, compared to the non-treated cells at each time point.

Further evaluation of the anti-proliferation mechanism exhibited by SN-F11/12 on MDA-MB-231 was performed using the PCR technique, where the determined constant EC50 value was used to treat MDA-MB-231 cells and subsequently the expression of two proliferative markers, Ki67 and PCNA, were examined. Beta actin was used as the loading control. The expression of Ki-67 and PCNA was found down-regulated compared to the cells grown without treatment (Figure 4a). Ki-67 expression was reduced significantly in MDA-MB-23 cell after 36 and 48 hours treatment with SN-F11/12 compared with the un-treated cells. The expression of PCNA between treated and un-treated cell was not significantly different although the SN-F11/12 treated cells expressed lower levels of PCNA (Figure 4b).

**Figure 5:**
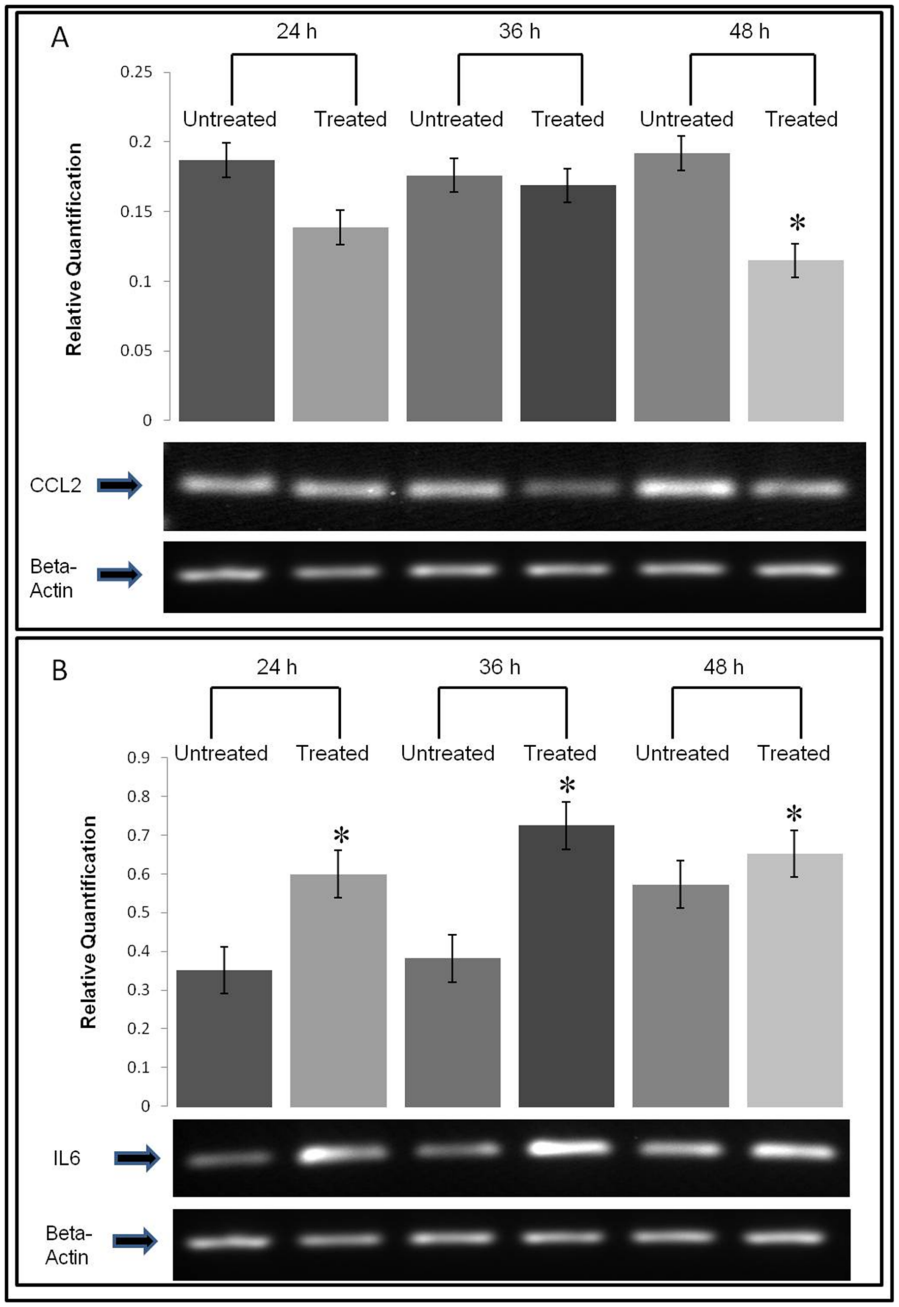
The expressions of invasion markers on the SN-F11/12-treated MDA-MB-231 cells. The figures show normalised mRNA expression of (A) CCL2 and (B) IL6 on the treated MDA-MB-231 cells. The target gene mRNA expression was normalised to the mRNA expression of beta-actin. Each data represents mean ± SE from three independent experiments. Statistical analysis was determined using the Student’s *t* test with *p<0.05 as statistical significance, compared to the non-treated cells at each time point.

Apart from the proliferative markers, the expression of two invasion markers, CCL-2 and IL-6 were also examined using the PCR technique. The expression of CCL2 in MDA-MB-231 cell was down-regulated after SN-F11/12 treatment as compared to the un-treated cell (Figure 5a). On the contrary, the expression of IL-6, which is usually involved in promoting cell invasion, was found up-regulated following SN-F11/12 treatment (Figure 5b).

**Figure 6:**
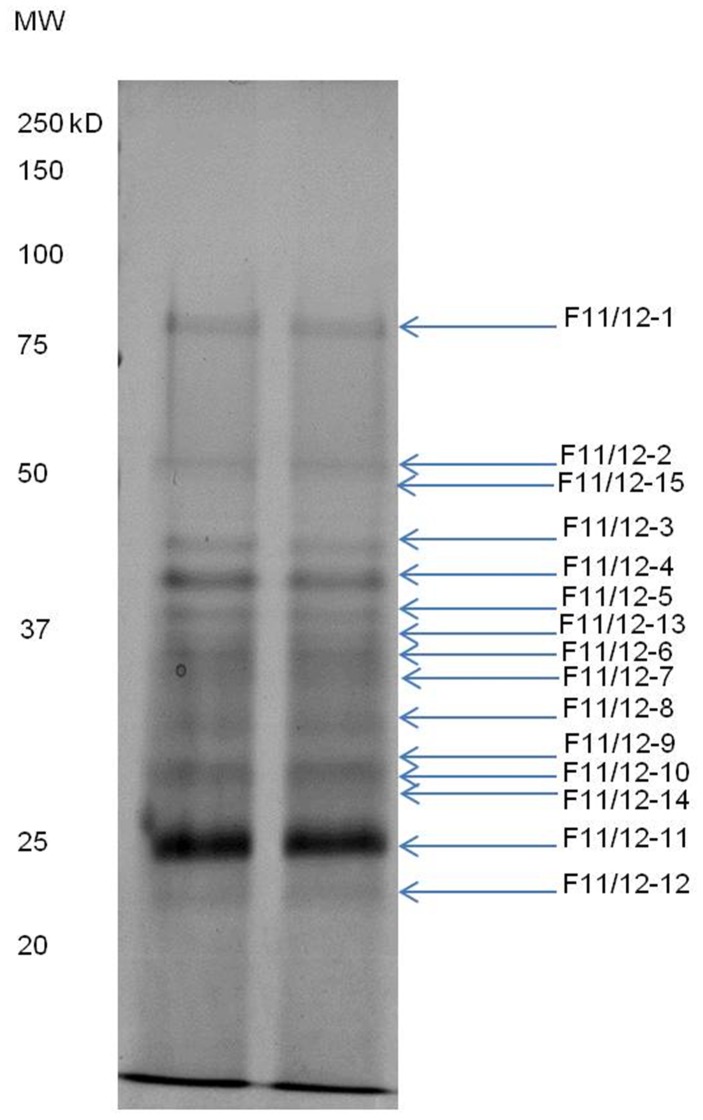
The protein profile analysis of SN-F11/12 by SDS-PAGE. The identified protein bands were numbered as indicated on the right. The analysis identified 15 protein bands on the gel.

SDS-PAGE protein profiling on SN-F11/12 showed the presence of 15 protein bands, each band was excised for in-gel digestion and the tryptic-digested peptides were analyzed using LC/Q-TOF. Figure 6 shows SDS-PAGE profile of the active fraction SN-F11/12 and the protein identified was listed in Table 1, 13 protein bands were successfully identified while 2 protein bands showed no protein hits.

**Table 1:** The list of identified proteins in the SN-F11/12.

Band	Identified Protein	Accession. No.	Score	Molecular Mass (kDa)	Sequence coverage (%)
SN-F11/12-1	Expressed protein	E1ZL32	180	51.4	2.46
SN-F11/12- 2	Malate dehydrogenase	O48904	172	35.9	11.37
SN-F11/12-3	No hit				
SN-F11/12-4	Malate dehydrogenase	O48904	201	35.9	4.66
SN-F11/12-5	Malate dehydrogenase	P86074	79	0.37	34.00
SN-F11/12-6	Malate dehydrogenase	O48904	306	35.9	24.78
	Putative uncharacterized protein At5g25340	Q8GXI1	131	23.5	27.88
SN-F11/12-7	Superoxide dismutase Cu Zn chloroplastic	O04997	668	22.2	8.64
	Malate dehydrogenase	O48904	315	35.9	17.78
SN-F11/12-8	Superoxide dismutase Cu Zn chloroplastic	O04997	112	22.2	5.00
SN-F11/12-9	Superoxide dismutase Cu Zn chloroplastic	O04997	376	22.2	15.45
	4 nitrophenylphosphatase	B6UCU8	266	39.2	17.91
	Predicted protein Fragment	F2DU72	169	34.9	10.22
	Ferredoxin NADP reductase leaf isozyme	B6TEW2	112	37.5	8.36
SN-F11/12-10	Malate dehydrogenase	O48904	266	35.9	17.78
SN-F11/12-11	Malate dehydrogenase	O48904	580	35.9	23.32
	Putative uncharacterized protein Fragment	F8PPI2	155	10.6	19.35
SN-F11/12-12	No hit				
SN-F11/12-13	Superoxide dismutase Cu Zn	O04996	644	15.4	19.61
	Predicted protein	F2D780	82	37.3	13.78
SN-F11/12-14	Ascorbate peroxidase	ABD97259	129	27.7	9.00
	Unknown	AFK33771	91	27.0	5.00
SN-F11/12-15	TIR-NBS-LRR class disease resistance protein	Q9FKE2	62	141.7	6.70

## Discussion

Previously, small molecular weight compounds from the leaf ethanolic extract and leaf aqueous extract of *G. procumbens* were shown to inhibit the growth of the breast cancer cell line T47D [9] and human mesangial cell proliferation [18], respectively. To date, there is no information on the bioactivity of protein extract from the leaf of *G. procumbens*. This is the first report revealing the cytotoxic property of the leaf proteins of *G. procumbens*. In this study, the leaf protein extracts of *G. procumbens* were subjected to a few purification steps before being used for testing. The extracts first underwent salt precipitation to remove small molecular weight compounds while the proteins were salted out as pellet. The pellet was shown to display cytotoxic activity on the MDA-MB-231 breast cancer cell line as observed under an inverted microscope. Subsequently, the pellet was subjected to purification by gel filtration chromatography for removal of salt and also for further separation of proteins according to size [17]. Following this, active protein fractions 11 and 12 were shown to have identical protein profiles and they were pooled as SN-F11/12 fraction. SN-F11/12 was found to exhibit potent cytotoxic activity on MDA-MB-23 breast cancer cell line as assessed by LDH cytotoxicity assay.

The data collected from the LDH cytotoxicity assay indicated that SN-F11/12 displayed potent inhibitory property on the growth of MDA-MB-231 cells with a constant EC50 value of 3.8 µg/mL. The anti-proliferative effect of SN-F11/12 was revealed by the suppression of the mRNAs of two proliferation markers, namely Ki67 and PCNA, that are essential for DNA replication [20]. Both the markers are cell cycle related genes that commonly used in the evaluation of *in vitro* anti-proliferative of drugs [19]. Ki67 is a nuclear antigen expressed in G1, G2 and M phases [20] while PCNA is expressed in G1, G2 and S phases [21] of cell cycle. Our results showed the differential expression of these two markers in SN-F11/12-treated and un-treated MDA-MB-231 cells, where the expression of Ki67 mRNA was significantly (p<0.05) down-regulated in the latter while the reduction of PCNA mRNA expression did not seem to differ significantly. Ki67 and PCNA have distinct characteristics and half-life [22]. In general, expression of Ki67 is solely indicating cell proliferation, whereas PCNA expression, besides cell proliferation, also indicates DNA repair or cell death. The observation of different levels of Ki67 mRNA and PCNA mRNA could be attributed to the half-life of PCNA, which is 20 times longer than that of Ki67. Moreover, PCNA protein is found post cell cycle and cell death. The detection of PCNA after cell death may be the reason for insignificant differential PCNA expression between SN-F11/12-treated and un-treated MDA-MB-231 cells. Therefore, the anti-proliferative effect of SN-F11/12 on MDA-MB-231 cells is likely correlated with Ki67 and PCNA, the commonly used proliferative indexes for *in vitro* study.

CCL2 and IL6 are involved in cancer cell invasion. In the process of cancer development, CCL2 together with macrophage-colony stimulating factor (M-CSF) and vascular endothelial growth factor (VEGF) play an active role to recruit circulating blood monocytes to the tumor site, where the recruited monocytes will be differentiated into tumor-associated macrophages (TAM) and establish a symbiotic relationship with the tumor cells. Subsequently, The TAM will secrete growth factors such as EGF, TGFβ, FGF, VEGF, IL1, TNF and IL6 that promote tumor cell proliferation, invasion and metastasis [23]. The mRNA expressions of the two invasion markers, CCL2 and IL6, were inversely to one another. The CCL2 mRNA expression was down-regulated while the IL6 mRNA expression was up-regulated in the SN-F11/12-treated MDA-MB-231 cells. CCL2 is one of the chemokines essential for breast cancer development and progression [24]. Overexpression of CCL2 promotes breast cancer metastasis [25]. On the contrary, inhibition of CCL2 and its receptor signaling pathway reduces metastasis *in vivo* and prolongs the survival of tumor-bearing mice [26]. IL6 is an interleukin that plays a key role in the pathophysiology of several cancers and various inflammatory disorders of the immune system [27]. IL6 over-expression has been implicated in the tumourigenesis of multiple myeloma, ovarian, renal cell, prostate, cervical and breast carcinomas [28], [29], [30], [31]. In a previous study, CCL2 was reported to contribute to the development of lung fibrosis by reducing IL6 levels [32]. In this study, treatment of MDA-MB-231 cells with SN-F11/12 decreased the expression of CCL2 mRNA in the cells. On the contrary, the expression of IL6 mRNA in the SN-F11/12-treated MDA-MB-231 cells was up-regulated when compared to the un-treated cells. As IL6 had been shown to be a pleiotropic cytokine with both tumor-promoting and inhibitory activity [33], the up-regulation of the expression of IL6 mRNA in the SN-F11/12-treated MDA-MB-231 cells may cause an inhibitory effect to the growth of the breast cancer cell line.

As SN-F11/12 has shown promising results of anti-proliferation and anti-invasion on MDA-MB-231 cells, the protein fraction was further evaluated by SDS-PAGE and mass spectrometry analysis. Out of the twelve proteins identified in SN-F11/12, two proteins, namely superoxide dismutase cooper zinc (Cu,Zn-SOD) and Toll Interleukin 1 Receptor-Nucleotide Binding Site-Leu-Rich Repeat (TIR-NBS-LRR) class disease resistance protein belonged to the plant defense proteins category. These plant defense proteins have also been detected in *Corydalis cava* tubers extract where they were found to inhibit the growth of HeLa cell line [34]. Superoxide dismutase (SOD) is an antioxidant that catalyzes the dismutation of superoxide anionic radicals to hydrogen peroxide and oxygen [35]. The protein belongs to the metalloenzyme group in which the activity relies on metal ion in the form of Cu,Zn-SOD, Mn-SOD and Fe-SOD [36]. Zhang *et al*. (2002) [37] reported that overexpression of Cu,Zn-SOD suppresses the growth of human tumor cells. However, Cu,Zn-SOD extracted from garlic displayed antagonist effect to doxorubicin, one of the most commonly used anti-cancer drugs, alleviates the anticancer drug cytotoxicity on tumor cell lines [38], [39]. In our present study, Cu,Zn-SOD was detected in four different protein bands of SN-F11/12. However, whether Cu,Zn-SOD is the protein that contributes to the anti-cancer property of the active protein fraction SN-F11/12 requires further evaluation.

The TIR-NBS-LRR class of disease resistance (R) proteins has been reported to play a crucial role in programmed cell death as part of the plant immune system [40]. The NBS domains exhibit ATPase activity while the LRR domain is known to mediate protein-protein and protein-ligand interactions. The TIR-NBS-LRR class of disease resistance (R) proteins is also involved in the specific recognition of pathogen-derived elicitor [41]. In the plant kingdom, the TIR domain that is present at the N-terminus of R proteins carries the NBS and LRR, while being necessary for cell death signaling [42].

Ascorbate peroxidase (APX) is a plant defense protein that was also detected in SN-F11/12. APX, together with glutathione peroxidase and catalase, make up the antioxidant enzymes that are involved in detoxification of hydrogen peroxide (H_2_O_2_). Following the formation of hydrogen peroxide through enzymatic reaction of SOD, detoxification of hydrogen peroxide is carried out by these antioxidant enzymes, which catalyze the conversion of hydrogen peroxide to water [43]. A balance ratio of SOD to the antioxidant enzymes is a required for the proper function of the cell, where high levels of SOD relative to the antioxidant enzymes will lead to an accumulation of superoxide radicals that are toxic to macromolecules, whilst low levels of SOD relative to the antioxidant enzymes will result in an increase in the concentration of hydrogen peroxide which will be subsequently converted to hydroxide radical (-OH), a very reactive species that is responsible for the oxidative damage of the cell [37].

Malate dehydrogenase was the most abundant protein detected in the active protein fraction of SN-F11/12, since it was detected in seven of the SDS-PAGE protein bands. Malate dehydrogenase catalyzes a reversible NAD^+^-dependent-dehydrogenase reaction in TCA cycle [44]. Kim *et. al* (2008) [45] revealed that the activity of malate dehydrogenase is antagonized by the activity of glucose 6-phosphate dehydrogenase in the dehydroepiandrosterone (DHEA) treated hepatocellular carcinoma rats. Glucose 6-phosphate dehydrogenase is the key enzyme regulating the first and irreversible step of pentose phosphate pathway which provides ribose-5-phosphate, a precursor for DNA synthesis in cell proliferation. The demand for ribose-5-phosphates for *de novo* synthesis of DNA has been shown to increase during the promotion phase of carcinogenesis [45]. Therefore the high abundance of malate dehydrogenase may cause a suppressive effect on the generation of ribose-5-phasphates in MDA-MB-231 cells which in turn leads to suppression of cell proliferation.

Ferredoxin NADP reductase and 4-nitrophenylphosphatase were also detected in the SN-F11/12. Ferredoxin NADP reductase is an enzyme involved in photosynthesis [46]. Mediavilla *et al.* (2010) [47] demonstrated that *Pisum sativum* ferredoxin NADP reductase is able to reduce the damages caused by hydrogen peroxide in Cos-7 cells. 4-nitrophenylphosphatase is an enzyme involved in p-nitrophenyl phosphate hydrolysis activity [48]. None of these proteins have been reported previously with their anti-cancer properties.

Other proteins detected in SN-F11/12 include six proteins that have not been identified yet. These proteins were registered as “expressed protein” (one protein), “uncharacterized proteins” (two proteins), “predicted proteins” (two proteins) and “unknown protein” (one protein). However, the functions of these proteins are not known.

The present study demonstrated that SN-F11/12 could be a potential anti-cancer agent although additional investigations need to be carried out to further evaluate its property. Nevertheless, since SN-F11/12 comprises of proteins, its oral consumption will not result in the desired bioactivity, as it will be absorbed by the digestive system. Furthermore, a protein-based drug will induce an humoral immune response in the host. Therefore, a good drug delivery system needs to be devised for its possible intended usage as a chemotherapy drug.

### Conclusion

The protein fraction, SN-F11/12, of *G. procumbens* was found to inhibit the growth of a breast cancer cell line, MDA-MB-231, at an EC_50_ value of 3.8 µg/mL. The down regulated expression of proliferation markers, Ki67 and PCNA, and invasion markers, CCL2 in the SN-F11/12 treated MDA-MB-231 cells reveals the possible route of cytotoxic mechanism of SN-F11/12. The potent anti-cancer property displayed by SN-F11/12 grants its potential use as a protein-based chemotherapeutic agent for breast cancer treatment.
